# Management of adverse events related to checkpoint inhibition therapy

**DOI:** 10.1007/s12254-018-0416-y

**Published:** 2018-06-12

**Authors:** Jakob Daniel Rudzki

**Affiliations:** 1 Medical University Innsbruck, Innsbruck, Austria; 2 UKIM V—Tirol Kliniken, Landeskrankenhaus Innsbruck, Anichstraße 35, 6020 Innsbruck, Austria

**Keywords:** Immunotherapy, Checkpoint inhibition, Immune-related adverse events, Guidelines, Recommendations

## Abstract

IO treatments (immuno-oncology treatments) have become reality and are now daily practice or, in some cases, a daily challenge. New recommendations are being made with the prime purpose of increasing alertness and awareness as well as emphasizing standard operating strategies to deal with immune-related adverse events (ir-AEs) in patients treated with immune checkpoint inhibitors (ICI). This brief review refers to systemic reviews, guidelines and meta-analyses, randomized controlled trials and case series published from 2000 to the present. Existing recommendations for optimal management of toxicities vary according to organ systems affected and grading. Grade 1 toxicities (exception to the rule: neurologic, hematologic, cardiac manifestation) require close monitoring. Grade 2 toxicities prompt immediate treatment interruption combined with corticosteroid administration (prednisone or methylprednisolone 0.5–1 mg/kg/day) until the symptoms revert to grade 1 or less. ir-AEs up to grade 3 or 4 justify suspension of treatment together with increased dosage of prednisone or methylprednisolone (1–2 mg/kg/day) combined with close monitoring to continuously adapt the current immunosuppressive strategy. In some cases, a different additional immunosuppressive agent has to be evaluated. Only when all symptoms have disappeared and immunosuppressive treatment produces a response can all immunosuppressive agents be tapered. Endocrinopathies are the exception to the rule and are mostly controllable by hormone replacement, at least in low-grade manifestation. This short review focuses on the main aspects that help manage immune-related side-effects and elucidates all the additional aspects surrounding and contributing to successful treatment and management of cancer patients.

## Immunotherapy and immune-related adverse events

The rise of the checkpoint blockade has been celebrated as the breakthrough of the year 2011. There is a wide diversity of checkpoint-inhibiting antibodies: anti-CTLA-4 (cytotoxic T‑lymphocyte-associated Protein 4; Yervoy® ipilimumab [1]), anti-PD‑1 (programmed cell death Protein 1; Opdivo® nivolumab [2, 3]; Keytruda® pembrolizumab [4]) or anti-PD-L1 directed against the ligand of PD‑1 (programmed death ligand 1; Bavencio® avelumab [5], Tecentriq® atezolizumab [6], Imfinzi® durvalumab [7]), all of which received approval from the US Food and Drug Administration (FDA) and some from the European Medicines Agency (EMA) for treatment of diverse entities of malignancies (metastatic melanoma, metastatic Merkel cell carcinoma, squamous and nonsquamous lung cancer, bladder cancer, metastatic renal cell cancer, therapy-resistant Hodgkin’s lymphoma, head and neck cancer, hepatic cell cancer, mismatch repair-deficient/microsatellite instability-high solid tumors). While these have to be mentioned, the brevity of this overview precludes their discussion.

Treatment of metastatic melanoma and both entities of lung cancer (squamous and nonsquamous cell carcinoma) mainly contributed to the success of ICIs as part of current treatment protocols. While in many cases sustained antitumor response directly correlates with clinical benefit, it may be associated with a wide spectrum of adverse events (presented at the American Association for Cancer Research 2017 Annual Meeting in Washington and commented by Julie Brahmer: Abstract No.: CT077, on 3 April 2017; Checkmate 209-003 ph1b study: 5a OS for the overall study population: 16%, 95% CI 10–23. Survival was seen across PD-L1 expression levels and tumor histologies, but with the bias of not being randomized, blinded or controlled). The reported AEs mainly differ from all known side-effects generated by cytotoxic therapies (chemotherapy, radiotherapy) or antibodies or combinations of them. A wide range of organ systems can be affected (skin, gastrointestinal tract, lungs, endocrine, nervous: peripheral as well as central, ocular, cardiovascular system). The occurring inflammatory reactions strongly resemble autoimmune disorders. While severity is generally mild, even life-threatening complications have been detected. There are some differences between anti-CTLA-4 and anti-PD1/-PDL1 antibodies, especially in grading and duration of occurrence of ir-AEs [8, 9]. There is no direct correlation to drug administration, with infusion related reactions (IRR) documented in very rare cases, namely fewer than 1% of patients. ir-AEs occur even after treatment has been suspended for weeks or even months and this has to be kept in mind when managing patients receiving IO (immuno-oncology) treatment [8].

## Recognition is the first step to successful treatment

Many very interesting reviews provide extensive information and elaborate this topic in a concise manner [10–12].

Successful management of ir-AEs irrespective of the affected organ systems demands that the patient, family caregivers and the patient’s GP (general practitioner) be kept abreast of the latest IO developments. All of these persons need to be informed about the IO concept and the clinical profile of possible AEs before the concept is initiated, and their information level should be maintained throughout the treatment period. The aim is to generate a “sound” but high level of suspicion for the fact that new symptoms occurring during and even after treatment start may be primarily related to ICI treatment. Grading of toxicities follows CTCAE version 5.0 (National Cancer Institute: Common Terminology Criteria for Adverse Events [CTCAE] 5.0.).

## Recommended management of ir-AEs

An increasing number of treatment approaches using ICIs require early recognition of symptoms in connection with changes in laboratory results. Every new symptom must be viewed in connection with the treatment and regarded as an ir-AE until proven otherwise. Diagnostic tools (laboratory results, body scans) are helpful in excluding ir-AEs. Identifying patients at higher risk for ir-AEs demands prompt and aggressive reaction. Recent data suggest that patients suffering from autoimmune diseases are eligible for checkpoint inhibition although they are currently excluded from all trials. There are ways to treat them safely while remaining more alert for unexpected adverse events, although they are still seriously underrepresented [13] and could form a group of high-risk patients. Indeed, such patients do not show a higher incidence of ir-AEs despite having active disease. Hence, treatment-specific risk factors are defined by IO/IO combinations and by the checkpoint inhibitor itself due to differences in incidence and severity of ir-AEs between anti-CTLA-4 [1], anti-PD1 [2–4], and anti-PDL1 antibodies [6, 7].

A heightened awareness for early recognition and treatment helps to mitigate the severity of ir-AEs.

## Organ-specific recommendations for ir-AEs

In most cases (with some exceptions) ir-AEs grade 2 can be managed by promptly interrupting treatment and providing supportive care. Questionnaires or standardized assessments assist in early recognition. New symptoms or changes in a patient’s health indicate early onset of ir-AEs. Giving patients wallet cards containing detailed information about symptoms and the patient’s immunotherapy history (documented ir-AEs with grading) ensures that all care providers (GPs but also emergency department staff) have additional information to handle the situation and avoid morbidity and ultimately even mortality.

In the case of grade 1 toxicities, watchful continuation of the IO treatment is generally appropriate. However, without complete resolution of symptoms or deterioration to grade 2 or worse immunosuppressive treatment must be started at 0.5 to 1 mg/kg/day prednisone or equivalent corticosteroid combined with tapered discontinuation of treatment. Such tapering of systemic corticosteroids over 4–6 weeks is performed contingent on resolution of the individual’s immune-related reaction. Grade 3 or 4 ir-AEs involve some substantial differences concerning the affected organ system. These differences are discussed in the subsequent part of this article and summarized in Table 1 (pointing out the management of severe adverse events = CTCAE ≥ grade 3 in defined organ toxicities).

**Table 1:** Management of ir-AEs in patients treated with ICIs/overview of the most relevant (S)AEs (severe adverse events) focused on severe adverse events (CTCAE 5.0 ≥ 3). (Adapted from [10]; based on expert consensus based recommendations with benefits outweighing harms—strength of recommendations is moderate)

	Grading	Management
**1. Skin toxicities**	*According to CTCAE is a challenge for skin. Severity is based on BSA, tolerability, morbidity and duration*	
**1.1. Rash/inflammatory dermatitis** *Erythema multiforme minor, lichenoid, eczematous*	G3: as G2 but with failure to respond to indicated interventions for G2 dermatitis	Holding ICI & weekly monitoring and treat with topical emollients, antihistamines & high-potency topical corticosteroids + initiate 1–2 mg/kg corticosteroid^a^ + tapering over at least 4 weeks
	G4: all severe rashes unmanageable with prior interventions	Systemic corticosteroids IV ^a^ 1–2 mg/kg with slow tapering when toxicity resolves. Consult with dermatology. Consider alternative antineoplastic therapy, if ICIs are the patient’s only option, consider restarting once these adverse effects have resolved to a G1 level
**1.2. Bullous dermatoses** *including bullous pemphigoid or other autoimmune bullous dermatoses, bullous drug reaction*	G3: skin sloughing covering >30% BSA with associated pain and limiting self-care ADL	Hold ICI & consult with dermatology; administer IV corticosteroids^a^ 1–2 mg/kg, tapering over at least 4 weeks, if bullous pemphigoid is diagnosed, it may be possible to avoid long term use of systemic corticosteroids and start with rituximab as an alternative approach to treat ir-AEs. Seek infectious disease consultation if patient have secondary cellulitis or other infection risk factors like neutropenia etc.
	G4: blisters covering >30% BSA with associated fluid or electrolyte abnormalities	Permanently discontinue ICIs, place patient under supervision of a dermatologist, administer IV corticosteroids^a^ 1–2 mg/kg & treat with rituximab as an alternative approach + seek infectious disease consultation if patient have secondary cellulitis or other infection risk factors like neutropenia etc.
**1.3. SCARs (including SJS, TEN, acute generalized exanthematous pustulosis, DRESS/DIHS)** *severe changes in either structure or function of skin, the appendages or the mucous membranes due to a drug*	G3: skin sloughing covering <10% BSA with mucosal involvement associated signs (e.g., erythema, purpura, epidermal detachment, mucous membrane detachment)	Hold ICI & consult with dermatology; treat skin with topical emollients & other petrolatum emollients; administer IV corticosteroids^a^ 0.5-1 mg/kg & convert to oral on response, wean over at least 4 weeks; add additional immunosuppressive agent (cyclosporine) in corticosteroid unresponsive cases
	G4:skin erythema & blistering/ sloughing covering ≥10 to >30% BSA with associated signs (e.g., erythema, purpura, epidermal detachment, mucous membrane detachment) and/or systemic symptoms and concerning associated blood work abnormalities (e.g., liver function test elevations in the setting of DRESS/DIHS)	Permanently discontinue ICIs; admit patient immediately to a burn unit or ICU with consulted dermatology & wound care services. Initiate IV corticosteroids^a^ 1–2 mg/kg and tapering when toxicity resolves to normal; IVIG or cyclosporine may also be considered in corticosteroid unresponsive cases
*Additional considerations: the usual prohibition of corticosteroids for SJS is not relevant here, as the underlying mechanism is a T-cell immunodirected toxicity. Adequate suppression is necessary with corticosteroids or other agents and may be prolonged in cases of DRESS/DIHS*		
**2. Enterocolitis**		*Work up of blood; testing of lactoferrin (to determine who needs more urgent endoscopy) & calprotectin (to follow-up on disease activity); screening laboratories (HIV* *, hepatitis A & B, blood quantiferon for tuberculosis) to prepare patients for infliximab treatment & should be routinely determined in patients at high risk; imaging (e.g., CT scan of abdomen & pelvis & GI endoscopy with biopsy) for early detection of ulceration in the colon that can predict a corticosteroid-refractory course that may require early infliximab treatment*
	G3: increase of 7 or more stools per day over baseline, incontinence, hospitalization indicated, severe increase in ostomy output compared with baseline, limited self-care ADL	Consider permanently discontinuing CTLA-4 agents and may restart PD-1, PD-L1 agents if patient can recover to G1 or less., administer corticosteroids^a^ 1–2 mg/kg; if symptoms persist ≥3–5 days or recur after improvement, consider administering IV corticosteroids^a^ or infliximab; consider colonoscopy in cases where patients have been on immunosuppression & may be at risk for opportunistic infections (i.e., CMV colitis) & for those who are anti-TNF or corticosteroid refractory
	G4: life-threatening situation; urgent intervention indicated	Permanently discontinue treatment; administer 1–2 mg/kg corticosteroids^a^ until symptoms improve to G1 & then start taper over 4–6 weeks; if symptoms are refractory to corticosteroids administer infliximab 5–10 mg/kg within 2–3 days. If there is concern of new infection or symptoms remain refractory to treatment consider lower GI endoscopy and consider vedolizumab in patients refractory to infliximab and/or contraindicated to TNF-alpha blockade.^b^
**3. Hepatitis**	G3: symptomatic liver dysfunction, fibrosis by biopsy, compensated cirrhosis, reactivation of chronic hepatitis (AST or ALT 5–20 × ULN and/or total bilirubin 3–10 × ULN)	Permanently discontinue ICIs, immediately start corticosteroid^a^ 1–2 mg/kg; if no improvement assessed after 3 days or if corticosteroid refractory, consider mycophenolate mofetil or azathioprine, daily laboratories & close monitoring for patients with AST/ALT > 8 × ULN and or elevated TB 3 × ULN (infliximab should not be used—potential risk of liver failure); corticosteroid taper can be attempted around 4–6 weeks; re-escalate if needed, optimal duration is unclear
	G4: decompensated liver function (e.g., ascites, coagulopathy, encephalopathy, coma; AST or ALT > 20 × ULN and/or total bilirubin >10 ULN)	Permanently discontinue ICIs, immediately start corticosteroid^a^ 2 mg/kg, if no improvement assessed after 3 days or if corticosteroid refractory the same management as in the case of G3
**4. Pneumonitis** *focal or diffuse inflammation of the lung parenchyma (typically identified on CT imaging)*	G3: severe symptoms, hospitalization required, involves all lung lobes or >50% of lung parenchyma, limiting self-care ADL, oxygen indicated	Permanently discontinue ICI, empirical antibiotics; administer corticosteroid^a^ 1–2 mg/kg, no improvement after 48 h, add infliximab 5 mg/kg or mycophenolate mofetil IV 1 g twice a day or IVIG for 5 days or cyclophosphamide; taper corticosteroids over 4–6 weeks. Pulmonary & infectious disease consults if necessary, BAL ± transbronchial biopsy offered
	G4: life threatening respiratory compromise, urgent intervention indicated (intubation)	
**5. Endocrinopathies**		
**5.1. Primary hypothyroidism**	G3–4: severe symptoms, medically significant or life-threatening consequences, unable to perform ADL	Hold ICI until symptoms resolve to baseline with appropriate supplementation; endocrine consultation, add IV therapy if signs of myxedema (bradycardia, hypothermia); thyroid supplementation and reassessment as in G2
**5.2. Hyperthyroidism**	G3–4: severe symptoms, medically significant or life-threatening consequences, unable to perform ADL	Hold ICIs until symptoms resolve to baseline, endocrine consultation, beta blocker for symptomatic relief, for severe symptoms administer corticosteroids^a^ 1–2 mg/kg and taper over 1–2 weeks & consider also use of SSKI or thionamide (methimazole or PTU)
**5.3. Primary adrenal insufficiency**	G3–4: severe symptoms, medically significant or life-threatening consequences, unable to perform ADL	Hold ICIs until patient is stable on replacement hormone, endocrine consultation, IV stress dose corticosteroids on presentation (hydrocortisone 100 mg or dexamethasone 4 mg—if diagnosis is not clear & stimulation testing will be needed). Taper stress dose corticosteroids down to maintenance doses over 7–14 days after discharge, continue maintenance therapy as in G1 and titrate down as symptoms dictate
**5.4. Hypophysitis** *Low ACTH with low cortisol; low or normal TSH with a low FT4; hypernatremia and volume depletion with diabetes insipidus; low testosterone or estradiol with low LH & FSH*		*Consider MRI of the brain with or without contrast with pituitary/sellar cuts in patients with multiple endocrine abnormalities* *±* *new severe headaches or complaints of vision changes*
	G3–4: severe symptoms, medically significant or life-threatening consequences, unable to perform ADL	Hold ICIs until patient is stable on replacement hormone; hormonal replacement as in G1; consider initial pulse therapy with corticosteroid^a^ 1–2 mg/kg oral daily with subsequent tapering over at least 1–2 weeks
**5.5. Diabetes** *T1DM (autoimmune) results from islet destruction and is often acute onset, with ketosis and an insulin requirement*	G3–4: severe symptoms, medically significant or life-threatening consequences, unable to perform ADL G3: >250–500 mg/dl	Hold ICIs until glucose control is obtained on therapy with reduction of toxicity to G1 or less, urgent endocrine consultation for all patients, admit for inpatient management: possibility of developing DKA
	G4: >500 mg/dl	
**6. Nervous system toxicities**		
**6.1. Myastenia gravis**	G3–4: limiting self-care and aids warranted, weakness limiting walking, any dysphagia, facial weakness, respiratory muscle weakness or rapidly progressive symptoms	Permanently discontinue ICIs; admit patient, may need ICU level monitoring, neurology consult, continue corticosteroids^a^ 1–2 mg/kg and initiate IVIG 2 g/kg IV over 5 days (0.4 g/kg/day) or plasmapheresis for 5 days, frequent pulmonary function assessment, daily neurologic review. Pyridostigmine starting at 30 mg orally three times a day and gradually increase based on symptoms
**6.2. Guillain-Barré syndrome** *Corticosteroids are usually not recommended for idiopathic Guillain-Barré syndrome, however, in ICIs related forms a treatment is reasonable*	G3–4: severe, limiting self-care and aids warranted, weakness limiting walking, any dysphagia, facial weakness, respiratory muscle weakness or rapidly progressive symptoms	Rapid transfer to ICU-level monitoring, start IVIG (0.4 g/kg/day for 5 days) or plasmapheresis; administer corticosteroids^a^ 2–4 mg/kg/day followed by slow taper, frequent pulmonary function assessment, daily neurologic review; nonopioid management of neuropathic pain
**6.3. Peripheral neuropathy**	G3–4: severe, limiting self-care and aids warranted, weakness limiting walking; severe may be Guillain-Barré syndrome	Proceed as per Guillain-Barré syndrome
**6.4. Autonomic neuropathy**	G3–4: severe limiting self-care and aids warranted	Permanently discontinue ICIs; admit patient; initiate corticosteroid^a^ 1 g daily for 3 days followed by taper, neurologic consultation
**6.5. Aseptic meningitis**	G3–4: severe, limiting self-care and aids warranted	Hold ICI and discuss resumption with patient only after taking into account the risks and benefits. Consider empirical antiviral (IV acyclovir) and antibacterial therapy until CSF results—once bacterial & viral infection are negative, may closely monitor off corticosteroids^a^ or consider oral corticosteroids^a^ 0.5 –1 mg/kg/day if moderate/severe symptoms
**6.6. Encephalitis**	G3–4: severe, limiting self-care and aids warranted	Proceed as per aseptic meningitis, consider administration of corticosteroid^a^ 1–2 mg/kg if severe: pulse corticosteroid^a^ 1 g IV for 3–5 days plus IVIG 2 g/kg over 5 days; if positive for autoimmune encephalopathy antibody and limited or no improvement, consider rituximab or plasmapheresis in consultation with neurology
**6.7. Transverse myelitis**	G3–4: severe, limiting self-care and aids warranted	Permanently discontinue ICIs; administer corticosteroid^a^ 2 mg/kg; strongly consider higher doses of 2 g/day for 3–5 days; strongly consider IVIG
**7. Myocarditis, pericarditis, arrhythmias, impaired ventricular function with heart failure and vasculitis**	G3: moderately abnormal testing or symptoms with mild activity	Hold ICIs and permanently discontinue after G1
	G4: moderate or severe decompensation; IV medication or intervention required, life-threatening conditions	High dose corticosteroids^a^ 1–2 mg/kg initiated rapidly (oral or IV depending on symptoms), admit patient, cardiology consultation; in patients without immediate response to high dose corticosteroids^a^, increase to 1 g every day of corticosteroids and consider the addition of either mycophenolate, infliximab or antithymocyte globulin
*Statement: Holding of checkpoint inhibitor therapies recommended for all grades of cardiovascular complications*		
**8. Nephritis**	G3: creatinine > 3 × baseline or > 4.0 mg/dl; hospitalization indicated	Permanently discontinue ICIs; administer corticosteroids^a^ 0.5–1 mg/kg, if worsening or no improvement increase to 1–2 mg/kg/day and taper over 4–6 weeks
	G4: life threatening consequences; dialysis indicated	Consult nephrology, evaluate for other causes; administer corticosteroids^a^ 1–2 mg/kg/day

*ACTH* adrenocorticotropic hormone, *ADL* activities of daily living, *ALT* alanine aminotransferase, *AST* aspartate aminotransferase, *BAL* bronchoalveolar lavage, *BSA* body surface area, *CSF* cerebrospinal fluid, *CTCAE 5.0* Common Terminology Criteria for Adverse Events, *CTLA‑4* cytotoxic T‑lymphocyte-associated Protein 4, *DIHS* drug-induced hypersensitivity syndrome, *DKA* diabetic ketoacidosis, *DRESS* drug reaction with eosinophilia and systemic syndrome, *FT4* free thyroxine, *G* grade, *HIV* humane immunodeficiency virus, *ICIs* immune-checkpoint inhibitors, *ICU* intensive care unit, *IV* intravenous, *IVIG* intravenous immunoglobulin, *MRI* magnetic resonance imaging, *PD‑1* programmed cell death Protein 1, *PD-L1* programmed death ligand 1, *PTU* propylthiouracil, *SCARs* severe cutaneous adverse reactions, *SJS* Stevens-Johnson syndrome, *SSKI* potassium iodide, *TB* total bilirubin, *TEN* toxic epidermal necrolysis, *TNF alpha* tumor necrosis factor alpha, *T1DM* type 1 diabetes mellitus, *TSH* thyroid-stimulating hormone, *ULN* upper limit of normal

^a^(Methyl)prednisolone or equivalent

^b^This decision should be made on an individual basis and is based on case series showing promising results

## Skin toxicities

In the case of failure of high-dose corticosteroid treatment (up to 2 mg/kg/day) an additional immunosuppressive therapy regimen is available: rituximab 375 mg/m^2^ is recommended instead of high-dose corticosteroids for bullous dermatoses and cyclosporine with or without IVIG (intravenous immunoglobulin) for severe or steroid-resistant cases of SCARs (severe cutaneous adverse reactions) including SJS (Stevens–Johnson syndrome), TEN (toxic epidermal necrolysis), acute generalized exanthematous pustulosis, and DRESS (drug reaction with eosinophilia and systemic syndrome)/DIHS (drug-induced hypersensitivity syndrome).

## Enterocolitis

Furthermore, grade 3 or 4 enterocolitis is mainly documented in IO/IO combination approaches (anti-CTLA-4 plus anti-PD-1 antibodies) illustrated in checkmate 063 in advanced melanoma in up to 13% of patients [14]. However, even the sole administration of ICIs results in severe colitis in fewer than <1% of patients, as shown in checkmate 037 and checkmate 066 (both melanoma trials using nivolumab) [15] and checkmate 017 in SQ-NSCLC (nivolumab) [3] and mentioned in treatment with anti-CTLA-4 antibody. This correlates with long-lasting responses persisting even after discontinuation of IO therapy [16]. In the case of grade 3 or 4 ir-AEs early administration of infliximab 5–10 mg/kg must be weighed if there is no response. Therefore, screening laboratory work (like HIV, hepatitis A & B, blood quantiferon for tuberculosis) must be routinely done in patients at high risk in order to be prepared to start infliximab, if and when necessary. Administration of vedolizumab (anti-integrin antibody) is an option in patients who are refractory to infliximab and/or contraindicated for TNF-alpha blockade. Nonsteroidal anti-inflammatory drugs (NSAIDs) are reported to be associated with an increase in ICI-induced enterocolitis [17].

## Hepatitis

Hepatitis (grade 3: AST/ALT 5–20 × ULN and/or total bilirubin 3‑10 × ULN) needs a second immunosuppressive agent like mycophenolate mofetil 1 g bid, which should be started if no improvement is assessed after 3 days.

## Pneumonitis

Worsening to grade 3 ir-AE always demands permanent discontinuation of ICI, escalated prednisone 1–2 mg/kg/day IV and, if no improvement is observed, the addition of infliximab, MMF or IVIG over 5 days or cyclophosphamide. In life-threatening grade 4 pneumonitis urgent intervention is needed with empirical antibiotics and bronchoscopy with BAL ± transbronchial biopsy if there are any doubts concerning the diagnosis. Less severe pneumonitis (grades 1–2) can be treated with oral prednisone 1–2 mg/kg/day whenever the patient is clinically stable and eligible for regular outpatient visits.

## Endocrinopathies

In many cases, endocrine dysfunction (hypothyroidism, hyperthyroidism, adrenal insufficiency, hypophysitis) can be adequately treated on a symptomatic basis except in patients with diabetes, where such treatment could trigger a life-threatening situation as T1DM (type 1 diabetes mellitus) with ketoacidosis demands a strict work-up and insulin substitution.

Cranial MRI (with or without contrast) is indicated for complaints associated with vision changes, severe headaches and multiple endocrine abnormalities (including alterations in diverse electrolytes) in order to exclude hypophysitis [4].

## Nervous system toxicities

Neurologic disorders like myasthenia gravis, Guillain–Barré syndrome, peripheral neuropathy, autonomic neuropathy, aseptic meningitis, encephalitis and transverse myelitis are treated with 1–2 mg/kg/day prednisone (grade 3) and, if there is no steroid response, IVIGs or plasmaphereses are ultimately alternative options.

## Myocarditis

Cardiovascular complications (myocarditis, pericarditis, arrhythmias, impaired ventricular function with heart failure and vasculitis) require primary administration of corticosteroids. Furthermore, if grading deteriorates (grade 3 or 4) and/or there is no response to corticosteroid, treatment must be adapted by adding mycophenolate mofetil, infliximab or ATG (antithymocyte globulin).

## Future aspects and remarks

In the context of ir-AEs, which vary in grading and duration of occurrence, manifestation always correlates with progression-free survival (documented in ongoing and already completed IO studies). Taking into account the fact that responders are primarily long responders, oncologists should maintain a sound awareness for the possibility of evolving immune-related adverse events of diverse grading [10] in order to be able to react promptly and correctly to keep their patients on ICI therapy as long as possible. Therefore, as so often in life, good timing can make all the difference.
